# The SAFE-study: comparative treatment effect, side effect and health evaluation of allopathic versus ayurvedic treatments for major depressive disorder: a naturalistic real-world study

**DOI:** 10.3389/fpsyt.2026.1719639

**Published:** 2026-04-13

**Authors:** Bernhard Theodor Baune, Anusha Prabhakaran, Mansi M. Patel, Sarah Elisabeth Fromme Grübel, Jagdish Varma, Manishkumar Patel, Shivenarain Gupta

**Affiliations:** 1 Department of Psychiatry, University of Münster, Münster, Germany; 2 Department of Psychiatry, Maganbhai Adenwala Mahagujarat University, Nadiad, India; 3 Department of Psychiatry, Pramukhswami Medical College, Bhaikaka University, Anand, India

**Keywords:** allopathic psychiatry, ayurvedic psychiatry, day-to-day functioning, depression, quality of life, side effects, treatment effect

## Abstract

**Background:**

Major Depressive Disorder (MDD) is a prevalent mental health condition with diverse treatment modalities. There is an unmet need in the treatment of major psychiatric disorders such as depression and naturalistic treatment options which are desirable particularly for chronic and severe courses of mental illness. In addition, there is an evidence gap for naturalistic treatment options for depression. This study compares the treatment effects and side effects of allopathic and ayurvedic treatments for MDD within a real-world setting. Allopathic approaches typically involve antidepressants and psychotherapy, while ayurvedic treatments may encompass herbal remedies, panchakarma, manual therapies, dietary modifications, yoga, meditation and lifestyle interventions.

**Methods:**

This six-month, naturalistic observational study will involve 105 adult participants (18–65 years) diagnosed with MDD according to DSM-5 criteria. Participants will be categorized to one of three groups: allopathic-only treatment (n=35), ayurvedic-only treatment (n=35), or a combination of both (n=35). Depressive symptom severity will be assessed as primary endpoint using primarily the MDRS, and secondarily HAMD and PHQ-9 scales at baseline, every 2 weeks until week 8, followed by monthly assessments until months 6. Secondary outcomes will exploratively include side effects (UKU, GASE, MARS), quality of life and daily functioning (WHOQoL-BREF, GAF, FAST) as well as general health measures (e.g. lab tests).

**Results:**

The study will compare changes in primary and secondary outcome measures across the three treatment groups over time (baseline vs. week 8, month 3 and month 6) using appropriate statistical models for longitudinal data (e.g., repeated measures ANOVA, mixed-effects models.

**Conclusions:**

This study will provide valuable real-world evidence on the comparative treatment effects and side effects of allopathic and ayurvedic treatments for MDD, informing clinical practice and future research directions in this area. Findings will contribute to a more nuanced understanding of treatment options for individuals with MDD.

## Highlights

- There is an unmet need for evaluating naturalistic treatments of depression
- There is an urgent need to close evidence gaps for the evaluation of ayurvedic treatments for MDD
- SAFE compares allopathic and ayurvedic treatments for MDD within a real-world setting
- SAFE is a six-month study including 105 adults in three groups: allopathic, ayurvedic or a combination
- SAFE will provide evidence on the comparative treatment effects and side effects of allopathic and ayurvedic treatments for MDD

## 1 Introduction

### 1.1 Background and rationale

Major Depressive Disorder (MDD), a debilitating mood disorder characterized by persistent sadness, anhedonia, and impaired functioning, poses a significant global health challenge (1). While the precise aetiology remains complex and multifaceted, converging evidence points to a dysregulation of neurobiological systems interacting with environmental and psychosocial factors (2). This interplay underscores the need for comprehensive treatment approaches that address both the biological and psychological dimensions of the disorder (3). Current treatment strategies primarily utilize allopathic and, increasingly, complementary and alternative medicine (CAM) approaches, such as Ayurvedic medicine (4). Despite a large use of the global prevalence of use of CAM/Ayurveda in depression, there is an urgent need to make naturalistic treatments available and to address evidence and clinical gaps in the treatment of depression.

Allopathic treatments for MDD predominantly focus on pharmacological interventions and various forms of psychotherapy (5). Pharmacological treatments primarily involve antidepressants, targeting neurotransmitter systems to alleviate depressive symptoms (6). Selective serotonin reuptake inhibitors (SSRIs), serotonin-norepinephrine reuptake inhibitors (SNRIs), tricyclic antidepressants (TCAs), and monoamine oxidase inhibitors (MAOIs) represent the main classes of antidepressants, although their efficacy varies considerably between individuals. Side effects, including sexual dysfunction, weight gain, and sedation, can limit treatment adherence and overall effectiveness (7). Furthermore, a substantial proportion of individuals exhibit treatment resistance, necessitating alternative or augmentation strategies (8). Psychotherapeutic interventions, particularly cognitive behavioural therapy (CBT) and interpersonal psychotherapy (IPT), play a crucial role in modifying maladaptive thought patterns, improving coping skills, and enhancing interpersonal relationships (9). These therapies often demonstrate comparable efficacy to antidepressants, particularly in preventing relapse.

Ayurvedic medicine, an ancient holistic system originating from India, offers an alternative and complementary approach to MDD (10). This system emphasizes the interconnectedness of mind, body, and spirit, aiming to restore balance through various interventions. These include herbal remedies (e.g., Ashwagandha, Brahmi etc.), dietary modifications tailored to individual constitution (Prakriti), lifestyle adjustments promoting holistic well-being, and mind-body techniques such as yoga and meditation (11). Ayurvedic treatment is well established in India. It is a holistic whole system approach that traditionally consists of specific components (Panchakarma, manual therapies Dietary Modifications, Herbal Formulations, Lifestyle Modifications, Sattvavajaya and Daiva-vyapasraya-cikitsa) that are integrated into a common treatment approach. There are special hospitals that specialize in such holistic treatment concepts. Patients can choose whether they want to be treated in an allopathic or an Ayurvedic hospital. While level A (e.g. meta-analysis and RCTs) and level B (evidence from at least one high-quality study or multiple moderate-quality studies) evidence studies and some smaller studies suggest the potential efficacy of Ayurvedic treatments in alleviating depressive symptoms (12–14), robust, large-scale randomized controlled trials comparing Ayurvedic treatment to allopathic treatments are relatively scarce (15). Understanding the mechanisms through which Ayurvedic treatment exert their effects on mood and related symptoms remains an area of active research.

This necessitates a more comprehensive understanding of the relative treatment effects, side effects and potential synergistic effects of integrating allopathic and Ayurvedic treatments for MDD. A comparative evaluation of these approaches is crucial for informing evidence-based clinical practice and optimizing therapeutic strategies for individuals experiencing this pervasive and debilitating disorder. This study aims to address this critical gap in the literature through observation and analysis of real-world outcomes of allopathic and ayurvedic treatment modalities for MDD.

### 1.2 Hypothesis and objectives

Based on the published literature, we assume that allopathic and Ayurvedic treatments are equally effective in treating depression, both leading to improvements in symptom severity. Therefore, we do not expect any statistically significant difference in treatment effect between the treatment groups (between-group effect) in week 8. However, we hypothesize that both therapies will lead to an improvement in depression symptoms over a period of 8 weeks (within-group effect). The question is exploratory in nature due to the design and methodology.

This study will pursue the following scientific objectives:

1. Treatment Effect: To quantitatively compare the effect of allopathic and ayurvedic treatments (and their combination) in reducing the severity of depressive symptoms in MDD, as measured by validated rating scales (primary Montgomery-Åsberg Depression Rating Scale (MADRS), secondary Hamilton Depression Rating Scale (HAM-D) and Patient Health Questionnaire-9 (PHQ-9) at baseline, 8 weeks, 3 months and 6 months.
2. Side Effects and Quality of Life: To compare the incidence, type, and severity of adverse events associated with allopathic and ayurvedic treatments using standardized adverse event reporting like Udvalg for Kliniske Undersøgelser Scale (UKU), General Assessment of Side (GASE) and Medication Adherence Report Scale (MARS). Furthermore, to assess differences in quality of life (QoL) and daily functioning between treatment groups using validated QoL instruments (e.g., WHOQOL-BREF) and functional assessment scales (e.g., Global Assessment of Functioning (e.g., Global Assessment of Functioning (GAF) and Functional Assessment Short Test (FAST).
3. General Health Evaluation: To evaluate and compare changes in general health parameters (e.g., Body Mass Index (BMI), vital signs, relevant blood work indicators such as liver and kidney function tests, electrolytes, CRP, leukocytes, red blood cells, platelets, haemoglobin , haematocrit , red cell indices and TSH) across treatment groups over the study duration.

## 2 Methods

### 2.1 Trial design and study setting

**Figure 1:**
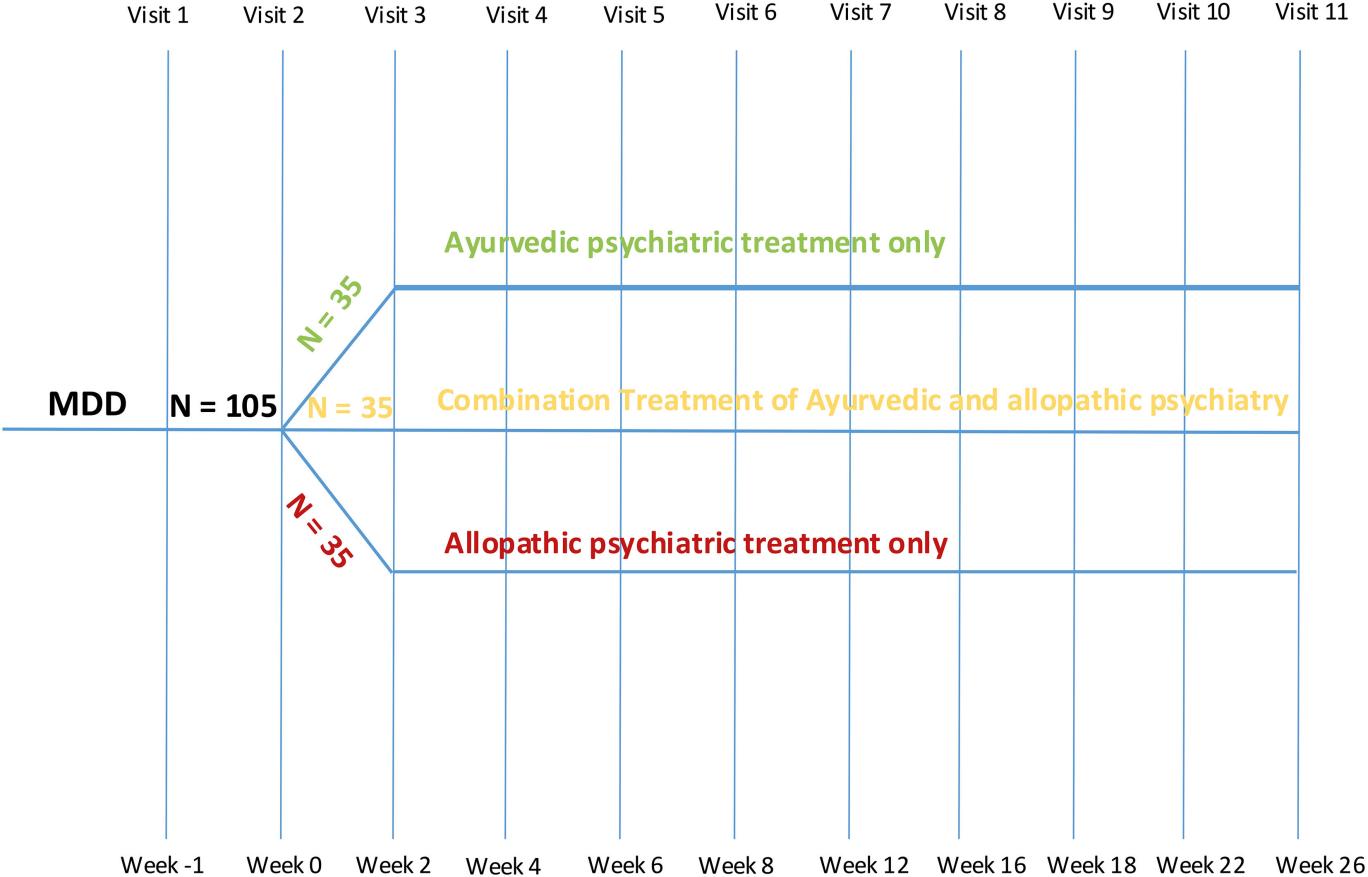
Flowchart of the study visits over time.

This study employs a naturalistic observational (non-randomization) design, conducted within real-world clinical settings for a six-month period (see **Figure 1**) where the group assignment reflects patient preference and clinical practice in specialized hospitals. The study population comprises 105 adult participants. Participants will be observed in one of three treatment groups, each consisting of approximately 35 individuals, based on the treatment they receive (see **Figure 1**).

- Arm A: Allopathic-Only Treatment: Participants in this arm will receive standard allopathic care, including antidepressant medication and psychotherapy as determined clinically appropriate.
- Arm B: Ayurvedic-Only Treatment: Participants in this arm will receive treatment exclusively with ayurvedic modalities.
- Arm C: Combination Treatment: This arm permits the integration of both ayurvedic and allopathic treatments, reflecting the increasing trend toward integrated care approaches.

At week 8 participants will be given the opportunity to reevaluate their treatment. This allows for a more patient-centred approach and accommodates individual responses to treatment. This enhances the study’s relevance to real-world clinical practice , where treatment decisions often involve adaptations based on ongoing assessment.

### 2.2 Study participants and eligibility criteria

#### 2.2.1 Inclusion criteria

To be eligible for participation in this study, potential participants must be adult females or males aged 18 to 65 years inclusive and be treated as in- and/or outpatients. Participants must have a primary diagnosis of MDD, of moderate or severe severity according to DSM-5 criteria. They need to agree to start a new bout of allopathic or Ayurvedic treatment in the current episode regardless of any previous treatments received during the current episode. They must provide informed consent to participate. They must also be willing to fully participate and adhere to the study protocol.

#### 2.2.2 Exclusion criteria

Individuals will be excluded from participation in this study if they have a current diagnosis of any severe psychiatric disorder other than MDD, such as bipolar disorder or schizophrenia. Participants with active suicidal ideation or behaviour , as assessed by the Columbia-Suicide Severity Rating Scale (C-SSRS) with a score of 4 or higher and with a moderate or severe alcohol use disorder within the past 12 months or any current alcohol or drug use disorder will be excluded. Pregnant or breastfeeding individuals will be excluded from the study to protect both the mother’s and the foetus ‘/infant’s health. Participants with significant medical or neurological conditions that could interfere with their ability to participate in the study or that may confound the interpretation of the results will be excluded. Participants who have significant language barriers that may hinder their understanding of study procedures or their ability to provide informed consent will be excluded.

### 2.3 Study intervention

This study will utilize a naturalistic observational design, leveraging the established expertise and practices of two distinct clinical settings: Nadiad Ayurveda Hospital (for Ayurvedic only and combined treatments) and Shree Krishna Hospital, Department of Psychiatry, Bhaikaka University, Karamsad, Anand (for allopathic treatments). The assignment to the groups follows a clear system. The two hospitals have different areas of focus. One clinic (Shree Krishna Hospital) provides exclusively allopathic treatment, while the other (Nadiad Ayurveda Hospital) provides Ayurvedic treatment. Patients who are treated at the Ayurvedic hospital but have previously received allopathic treatment will continue to receive allopathic treatment (combined therapy). The clinic is selected based on clinical indications and in consultation with the patient.

#### 2.3.1 Arm A: allopathic treatment arm

Participants assigned to the allopathic treatment arm will receive a comprehensive treatment plan developed and overseen by a licensed psychiatrist. This plan will adhere to current clinical practice guidelines for MDD and will include Pharmacological Intervention, Psychotherapy and Supportive Care.

A standard antidepressant medication regimen will be prescribed. The choice of antidepressant will be guided by evidence-based practice guidelines (Maudsley Prescribing Guidelines and Indian Psychiatric Society guidelines). Dosage adjustments will be made as needed based on clinical response and tolerability. Participants will also receive evidence-based psychotherapy, typically Cognitive Behavioural Therapy (CBT) or Interpersonal Psychotherapy (IPT), guided by a licensed mental health professional. The type and frequency of psychotherapy sessions will be tailored to each participant’s specific needs and clinical presentation.

Participants will receive supportive care as needed, which may include regular monitoring of their mental and physical health, strategies to enhance treatment adherence, and resources to address any practical or social challenges they may be facing.

#### 2.3.2 Arm B: ayurvedic treatment arm

Participants in the Ayurvedic treatment arm will receive an individualized treatment plan developed and overseen by a qualified Ayurvedic specialist. Treatment will be individualized, considering each patient’s unique constitution (Prakriti) and the imbalances (Vikriti) contributing to their MDD. This will involve a comprehensive assessment of physical and psychological symptoms, using traditional Ayurvedic diagnostic methods. This plan will be customized to each participant’s unique constitution (Prakriti) and will encompass Panchakarma, manual therapies Dietary Modifications, Herbal Formulations, Lifestyle Modifications, Sattvavajaya and Daiva-vyapasraya-cikitsa.

Depending on the individual’s need and condition, Panchakarma and manual therapies may include Vamana, Virecena, Bastis, Nasya, Shirodhara, Abhyanga, or other suitable treatments as determined by the Ayurvedic specialist. This will be administered by qualified Ayurvedic practitioners following standard Ayurvedic protocols and safety guidelines. Participants will receive personalized dietary advice aimed at promoting balance and well-being. The dietary recommendations will be tailored to their Prakriti and may involve specific food restrictions or recommendations depending upon their individual requirements and constitution. Participants may receive herbal formulations (e.g., Ashwagandha, Brahmi, Jatamansi, Tagara, Kapikacchu) selected based on their individual Prakriti and clinical presentation. The dosage and specific formulation will be determined by the Ayurvedic specialist. The herbal preparations will be sourced from reputable suppliers to ensure quality and purity. Lifestyle recommendations will be integrated into the treatment plan, emphasizing routines and practices that promote mental and physical health. This may include recommendations regarding sleep hygiene, stress management techniques, exercise regimes and the establishment of healthy daily habits. Participants may be recommended to incorporate Sattvavajaya and Daiva-vyapasraya-cikitsa into their daily routines. This may involve specific asanas and pranayama techniques, guided meditation practices, or other mind regulating interventions that support relaxation, stress reduction, and improved mental well-being.

#### 2.3.3 Arm C: combination treatment arm

Patients who are treated at the Ayurvedic hospital but have previously received allopathic treatment will continue to receive allopathic treatment and start new ayurvedic treatment. This arm integrates Allopathic and Ayurvedic treatments, collaboratively managed by both a Psychiatrist and an Ayurvedic specialist. The treatment strategy will be individualized, considering both the patient’s clinical presentation and preferences.

A patient who is already on Allopathic medication (antidepressants) from their psychiatrist and wishes to start ayurvedic treatment would be categorized into this group. The Ayurvedic specialist and the patients treating Psychiatrist would collaboratively decide whether to maintain, reduce, or discontinue the Allopathic medication as the Ayurvedic treatment progresses. If no Allopathic treatment has been initiated, Ayurvedic treatment will be administered for eight weeks before a joint clinical decision is made regarding the addition of allopathic medication. This approach aims to leverage the potential synergistic effects of both treatment modalities, promoting personalized and effective management of MDD. Previous studies have shown that allopathic psychiatric medication can be combined safely with Ayurvedic treatments such as herbs (10, 11, 16–18).

### 2.4 Study assessments

**Figure 2:**
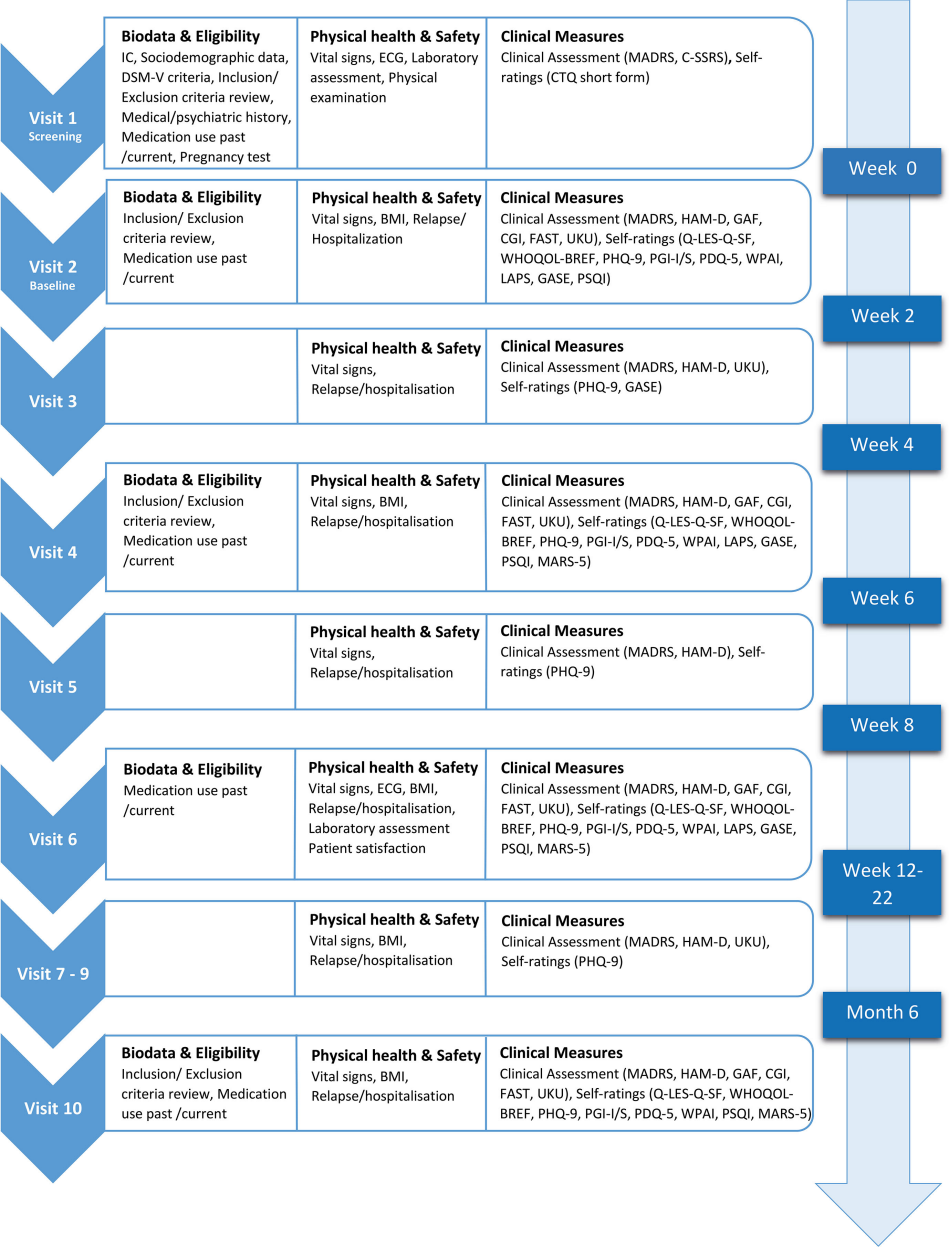
Overview of study procedures and study visit.

All three arms will involve structured assessments at baseline, every 2 weeks until week 8, followed by monthly assessments up to month 6 to measure symptom severity, quality of life, functional capacity, adverse events, and adherence (see **figure 2** for a detailed overview of assessment schedules across all visits). The study will involve a detailed medical history, including sociodemographics, medical and psychiatric history, and previous and current medication. Clinical symptoms related to depression, trauma, and sleep are assessed using the test procedures described below.

Ayurvedic data, i.e. prakṛti (constitution), agni (state of digestion and metabolism), koṣṭha (sensitivity of bowel), various parīkṣā (examinations) i.e. āturabala pramāṇa, jihvā, nādi, manaha parikṣā, nidāna pañcaka specifically for viśada and manas bhāva parīkṣā will be assessed of all the patients of Ayurveda group and combination group. These all will be assessed by using specific proforma. This proforma contains all above data in detailed as per the classical references. The recording of Ayurvedic parameters enables a comprehensive classification of the Ayurvedic definition of depression and the derivation of therapeutic measures.

#### 2.4.1 Description of study scales

Columbia-Suicide Severity Rating Scale (C-SSRS (19)) is a validated, standardized, and widely adopted tool for assessing suicidal ideation and behaviours, with strong predictive validity and global clinical and research applications.

The Montgomery-Åsberg Depression Rating Scale (MADRS (20)) is developed to diagnose depression. The ratings should be based on a clinical interview moving from broadly phrased questions about symptoms to more detailed ones which allow a precise rating of severity. The scale is reliable and valid and sensitive to change in the depressive symptoms.

The Hamilton Depression Rating Scale (HAM-D (21)) is a widely used clinician-administered instrument for assessing the severity of depressive symptoms in adults. It’s a structured interview-based scale that provides a quantitative measure of the patient’s current depressive state. The scores for all items are summed to produce a total score, which reflects the overall severity of depression. The most commonly used version is the 17-item version (HAM-D-17, REF).

The Patient Health Questionnaire-9 (PHQ-9 (22)) is a widely used self-report measure designed to assess the severity of depressive symptoms in adults. It’s a brief, reliable, and valid instrument frequently employed in clinical practice , research settings, and screening for depression. The PHQ-9 is a simple questionnaire where individuals rate the severity of each symptom on a four-point scale. The scores for each symptom are summed to produce a total score ranging from 0 to 27. Higher scores indicate greater symptom severity.

The Pittsburgh Sleep Quality Index (PSQI (23)) is a self-rated questionnaire designed to measure sleep quality and disturbances over a one-month period. Higher scores indicating poorer sleep quality.

The abbreviated version of the Childhood Trauma Questionnaire (CTQ-SF (24)) consists out of 28 items that describe situations while growing up. Items can be answered with “never true”, “rarely true”, “sometimes true”, “often true” or “very often true”. Bernstein et al. (24) tested this short version of the CTQ against the longer version and concluded that the scale demonstrated good criterion-related validity and that the same meaning across study samples was measured. To keep the time invested in the current study to a minimum, while obtaining all relevant data, the CTQ-SF was chosen. The average administration time is around 10 minutes.

The Leuven Affect and Pleasure Scale (LAPS (25)) is a 16-item scale in which treated subjects rate their positive and negative moods and hedonic tone from 0-10, where 0 is not at all, 1–3 are scores for a little bit, 4–6 are scores for moderately, 7–9 are scores for quite a bit and 10 represents very much.

The Patient Global Impression (PGI (26)scale (adapted from: CGI) Severity and Improvement is the (adapted) subject-rated version of the CGI. There are 2 questions: “On a scale from 1-7, where 1 is very much better and 7 is very much worse, how would you rate the improvement of your symptoms since the start of your new medication?” And: “On a scale of 1 to 7, where 1 is not at all ill and 7 is the worst that your illness has ever been, how would you rate the severity of your symptoms of depression?” The PGI scales are used in a broad range of disease.

In addition, there is a focus on assessing quality of life and everyday impairments. The test procedures used for this assessment are also briefly explained below.

Quality of Life Enjoyment and Satisfaction Questionnaire – Short Form (Q-LES-Q-SF (27)). The self-report questionnaire Q-LES-Q-SF consists of 14 items derived from the long form’s general activities subscale, plus 2 questions about medication and overall life satisfaction. Both versions are among the most frequently used quality of life measures in psychopharmacology and clinical trials, and have been translated into several languages. The Q-LES-Q-SF is a valid, reliable self-report instrument for assessing quality of life. To reduce the subject’s burden, we chose to use the short form of the Q-LES-Q-SF. The average administration time is around 5 minutes.

The WHOQOL-Bref is a short version of the World Health Organization Quality of Life (WHOQOL) assessment instrument (28). It’s a self-report questionnaire designed to measure quality of life across the four domains: Physical health, Psychological health, Social relationships and Environmental health. In addition to these four domains, the WHOQOL-Bref also includes a general assessment of overall quality of life and a section assessing overall health and functioning.

The Udvalg for Kliniske Undersøgelser scale (UKU (29)) is a brief, clinician-administered rating scale used to assess the severity of withdrawal symptoms. The scale focusses psychic, neurological, autonomic and other side effects.

The General Assessment of Side Effects (GASE (30)) consists of 36 items (symptom descriptions) organized by body parts. GASE collects information on symptoms experienced during the past week. These symptoms can be rated as “not present”, “mild”, “moderate,” or “severe.” In the current study, the subject rates the symptoms and the doctors rate the relatedness. The symptoms are noted as adverse event when the doctor rates the symptom as clinically significant. The GASE also requests to report symptoms not listed (spontaneous). When noted as adverse event, the doctor will also rate the severity and relatedness to medication. The GASE has high internal consistency, which indicates good reliability and is validated. The mean administration time is 5–10 minutes.

The Medication Adherence Report Scale – 5 items (MARS−5 (31, 32)) is a self−report questionnaire designed to assess both intentional and unintentional non-adherence to medication regimens. It is a condensed version of the original 10−item scale (MARS−10) developed by Rob Horne et al.

The Work Productivity and Activity Impairment (WPAI (33)) questionnaire is a self-report measure designed to assess the impact of health conditions on work productivity and activity impairment. It’s frequently used in clinical trials and research studies to quantify the effect of illnesses, including mental health disorders like depression, on various aspects of an individual’s work and daily life.

The Functional Assessment Short Test (FAST (34)) is a brief, clinician-administered instrument designed to assess the level of functional impairment in individuals with mental illness. It’s a useful tool for evaluating the impact of a mental health condition on various aspects of a person’s daily life, providing a quantitative measure of functional capacity. The FAST assesses functional impairment across the key domains Social Functioning, Occupational Functioning, Self-Care Functioning and Home-Living Functioning. A trained clinician administers the FAST, typically through an interview with the patient.

The Global Assessment of Functioning (GAF (35)) scale was a numerical scale used in psychiatry to rate a person’s overall level of psychological, social, and occupational functioning. Scores ranged from 0 to 100, with higher scores indicating better functioning and lower scores reflecting more severe impairment.

The Perceived Deficit Questionnaire (PDQ-5 (36)) was developed specifically for multiple sclerosis to provide a self-report measure of cognitive dysfunction. This instrument provides an assessment of several domains of cognitive functioning that are frequently affected in multiple sclerosis, as well as in psychotic and affective disorders: attention, retrospective memory, prospective memory, and planning and organization. From the full-length PDQ version, a short version with 5 items was created and validated. The PDQ-5 includes items from all 4 PDQ-20 subscales (attention/concentration, retrospective memory, prospective memory, planning/organization).

### 2.5 Data assessment

Data will be collected using a secure electronic Case Report Form (eCRF) within the REDCap platform. REDCap’s user-friendly interface and robust security features (hosted at the Department of Psychiatry, University of Münster, ensuring compliance with the General Data Protection Regulation (GDPR).

### 2.6 Standardisation and training of study team in study assessments

To ensure data quality, consistency, and adherence to standardized procedures, a comprehensive training program will be implemented prior to the commencement of any study activities. This training program will address all aspects of data collection, assessment, and treatment delivery. Training materials will include detailed manuals and practice sessions, with assessment of competency at the end of the training. To ensure accuracy in the diagnosis of Major Depressive Disorder (MDD), all diagnoses will be confirmed by experienced clinicians. A communication system will be implemented to facilitate seamless communication, data sharing, and decision making among the research teams at the three collaborating institutions.

The comprehensive training, diagnostic confirmation, and established teleconsultation procedures will enhance data quality, minimize bias, and maximize consistency in the conduct of this multi- centre study.

### 2.7 Primary and secondary outcome measures

The primary endpoint will focus on changes in depressive symptom severity, measured using primary MADRS, and secondary HAM-D and PHQ-9 especially in week 8. The use of multiple scales enhances the reliability and validity of the assessment, providing a more comprehensive picture of symptom change. Several secondary outcome measures will exploratively elucidate the impact of treatments on various aspects of patient well-being and functioning. These include side effects (UKU, GASE, MARS), quality of life and daily functioning (WHOQoL-BREF, GAF, FAST) as well as general health measures (e.g. lab tests). This comprehensive approach ensures that the study captures a broad range of outcomes, providing a robust and nuanced evaluation of the treatment effect and side effects of allopathic and Ayurvedic treatments for MDD. The question is exploratory in nature due to the design and methodology.

### 2.8 Ethical considerations and data protection

Ethical considerations will guide all aspects of the study, from design and implementation to data analysis and dissemination. The research protocol was approved by the relevant Institutional Ethics Review Boards (IERBs) at participating institutions (MAM University approval number: MAM UNI/IECHR/2025; Bhaikaka University approval number: IEC/BU/162/Faculty/25/135/2025). In addition, the study was pre-registered with the Indian authorities (CTRI/2025/07/090147).

All potential participants will receive comprehensive information about the study’s purpose, procedures, risks, and benefits ensuring that participants can make an informed decision about their participation. Written informed consent will be obtained from all participants prior to any study procedures being undertaken. Participants will be free to withdraw their consent and discontinue their participation at any time without penalty. Participants will be closely monitored for the occurrence of adverse events throughout the study period. Regular follow-up visits will allow for the detection and assessment of any adverse effects, whether related to the study interventions or other factors. If any adverse events occur medical attention, adjustments to the treatment regimen, counselling, or other supportive measures as needed appropriate interventions will be provided promptly. Serious adverse events will be reported immediately to the relevant IERBs and the study team. The study will have a clear protocol for managing adverse events. A data safety monitoring board will regularly review safety data to ensure participant safety. The study’s data management procedures will strictly adhere to relevant data protection regulations, which includes GDPR Compliance, Indian Data Protection Standards, Local Data Safety Standards and Data Anonymization.

### 2.9 Sample size calculation and statistical analysis

We hypothesise that all treatment groups lead to a statistically significant reduction in symptoms of depression between baseline and week 8 (within group effect). We also hypothesize that the groups do not differ in terms of treatment success (between group effect). We performed a power analysis to calculate the sample size. With a sample size of N = 105 patients, an alpha error probability of 0.5 and a moderate effect size (0.5) (for change in symptoms of depression), we achieve a statistical power of >0.8. An effect size of 0.5 equates to a mean difference in the MADRS score between baseline and week 8 of 50%, which reflects a commonly accepted clinical response measure in depression studies. In practise , this would mean a reduction of 15 points on the MADRS scale from 30 points at baseline to 15 points at week 8. Such a kind of reduction has been seen in other interventional studies in depression.

Data analysis will be conducted by the team at the Department of Psychiatry, University of Münster. The primary and secondary outcome measures will be analysed using an intention-to-treat (ITT) and exploratory approach. We will focus on specialized analyzation for longitudinal data. In addition, we will correct for confounding variables in all analyses where significant differences arise between the groups.

The choice of statistical test will depend on the nature of the data and the specific research question. For continuous outcome variables (e.g., scores on depression rating scales, quality of life scores, continuous physiological measures), Analysis of Variance (ANOVA) or independent samples t-tests will be used to compare means between the three treatment groups (Ayurvedic-only, Allopathic-only, and Combination). *Post-hoc* tests (e.g., Tukey’s HSD, Bonferroni correction) will be employed if significant differences are found between groups to determine which specific groups differ significantly. For categorical outcome variables (e.g., presence or absence of adverse events, categorical measures of quality of life, treatment adherence categories), Chi-square tests or Fisher’s exact tests (for small sample sizes) will be used to compare proportions between treatment groups. Given the repeated measures design (baseline, 8 weeks, 3 months, 6 months), appropriate statistical models for longitudinal data (e.g., repeated measures ANOVA, mixed-effects models) will be used to analyse changes in outcome measures over time within each treatment group and to examine the interaction effects between treatment group and time. This will allow for a more powerful examination of treatment effects and the assessment of treatment response trajectories. Strategies to address missing data will be carefully considered and documented. In addition to p-values, effect sizes (e.g., Cohen’s d) and 95% confidence intervals will be reported for all significant findings. All statistical analyses will be performed using appropriate statistical software packages (e.g., SPSS, R).

## 3 Discussion

Even though there are well-established and scientifically sound treatment approaches for depression, 30% of treatment cases are considered resistant to therapy (37, 38). These prevalence rates are worrying. There is an enormous need for alternative, low-side-effect, and effective complementary treatments for depression. Complementary and alternative medicine is widespread and established in both industrialized and developing countries. Up to 65% of people in industrialized countries and 80% of people in developing countries use it for psychosomatic complaints such as depression [42,43]. Studies that scientifically investigate complementary or alternative treatments focus predominantly on individual interventions, whereas Ayurveda is designed as a holistic treatment approach, which is a dominant feature of the study design (39).

### 3.1 Strengths

The study design we have developed provides an opportunity to evaluate allopathic, Ayurvedic, and combined therapeutic approaches in a real-world setting. The study also highlights the potential of integration of Ayurvedic and allopathic approaches as innovative approaches with real-world generalizability. The study is designed as a longitudinal study with a follow-up period of six months so that long-term effects can also be exploratively investigated. The study includes extensive clinical and somatic examinations, self-reports, and interviews so that both objective and subjective assessments can be considered. The design allows individualized and adaptive treatment of patients.

The problem of ergodicity (40)—that interindividual differences do not necessarily reflect intraindividual dynamics and that conclusions based on group-level data cannot be directly transferred to individual patients—plays a role in the interpretation of our findings. We explicitly use group-level results as evidence of average treatment effects and will use them as a starting point for individualized care. We operationalize this through measurement-based monitoring, stepwise treatment adaptation, and—where feasible—idiographic designs (e.g., intensive longitudinal assessments, single-case or N-of-1 approaches) to evaluate within-person responses and further optimize treatment for the individual patient.

The study thus offers high ecological validity and the potential to be transferable to everyday clinical practice . At the same time, the collection of routine blood markers and vital parameters offers a opportunity to gain exploratively neurobiological insights. The multi-professional team and international collaboration offer the advantage of combining in-depth expertise in various fields.

### 3.2 Limitations

While the real-world study has advantages for translation and generalizability to clinical practice , this comes with limitations such as potential confounding, selection bias, and heterogeneity of Ayurvedic and allopathic interventions, which could affect internal validity. In this context, it should be noted that the choice of treatment depends strongly on the preferences of the patients and the approach of the treating clinic and physicians, even though participatory decision-making processes are particularly important with regard to the treatment of psychiatric patients. Even though a wait-list control condition or a no-intervention arm would provide valuable data and improve the interpretability of the findings, this would not be ethically justifiable in our sample.

Moreover, the generalizability is limited to comparable settings in India. It should be noted that the exclusion of suicidality and substance abuse leads to a small but ethically necessary restriction of the naturalistic cohort.

Furthermore, the approach of a multimodal and routine (non-randomised) intervention program in both Ayurvedic and allopathic treatment arms have the potential disadvantage that no causal inferences can be made about specific effects of individual components of the treatment. However, we would like to emphasize that a more holistic approach to treating depression reflects clinical reality and carries a higher weight for translation into routine settings.

This study is designed to determine whether the applied treatments are effective in producing changes in the dependent variables. However, it is not intended to explain how or through which mechanisms the treatment leads to successful outcomes. Addressing this question would require a research design that captures the complexity of real-world settings with greater ecological validity (e.g., alternative approaches such as the integrative single-case design) (41).

## 4 Conclusions

With its comprehensive, methodologically sound, and realistic design, this study is positioned to generate valuable real-world evidence in a still young field of research. Rather than providing definitive clinical recommendations, the findings will serve as hypothesis-generating data on the potential effects of a holistic Ayurvedic approach, either alone or in combination with allopathic treatments, for individuals with depression. The results are expected to inform the design of future randomized controlled trials (RCTs) and highlight promising integrative strategies. In doing so, this study will stimulate further systematic investigation and contribute to the growing body of knowledge on integrative and complementary psychiatry.
